# Clinical Cases

**Published:** 1899-04

**Authors:** Wm. L. Morgan

**Affiliations:** Baltimore, Md.


### PROCEEDINGS OF THE SOCIETY OF HOMŒOPATHICIANS,

### BUREAU OF CLINICAL MEDICINE.

F. W. PATCH, *Chairman.*

### CLINICAL CASES.

BY WM. L. MORGAN, M. D., BALTIMORE, MD.

CASE 1, May 25th, 1898.—Freddie, age two years, son of the Rev. W., previously healthy. Has had cold, head and backache for several days past. Has now a fever, thirst, headache, sore throat, and aches all over; wants to eat, but is satiated as soon as he begins; full of wind, tongue coated, and right tonsil covered wtih a pale yellow membrane, with a membrane in fauces. The day before the family had moved from a very damp house with unsanitary surroundings and bad drainage. I did not take a culture for the city laboratory to pass judgment on, but gave Lyco., MM. and S. L. q. s. Next morning Freddie was well, and has remained well ever since.

CASES 2 AND 3, October 14th. 1898.—Mary and John D., aged four and six; bad sanitary condition of house. Mary had yellow membrane extending all over fauces and up into nose. She got Kali-bichro., CM. John had membrane on right tonsil, for which he got Lycop., CM. Both children were very sick at 9 P. M.

October 15th, 9 P. M., both children were well, and have remained well ever since. No cultures taken.

CASE 4, June 5th, 1897.—Miss L. L., age 17, well grown, good physique, rather pale, with a little yellowish tinge.

Had not yet menstruated. Always constipated.

Has hay fever every year on August 17th, continuing until cold weather. Eyes, nose, throat, ears, and genitals smart, burn, and itch, causing great distress all the time until relieved when the cold season sets in.

Taste and smell absent; can only taste acids.

Has alternate flushes of heat and sweat of the face.

Had milk-crust till seven years of age, which was "cured" with a tar salve. She has had hay asthma ever since, with wheezing in the chest.

She had grippe eighteen months ago; since then has had cough, mucus and sensation of dryness in throat, brow headache, dull pain across lower lumbar region, fever blisters on lips, ankles weak and often turn over when walking.

She got Nat-mur., CM. One dose, S. L., eight powders.

June 12th.—The grippe symptoms have all gone and she feels better in every way.

The mother described the milk-crust from memory as consisting of large scabs. On pressure thin pus would come out and run down on the face and neck, sometimes excoriating. This made me think of Graphites.

I gave her Graph. CM., and S. L.

July 6th.—Menses came on June 18th and lasted five days.

August 23d.—No hay fever on the 17th, and none to the present day.

I will not say whether it was the Nat-mur. or Graph. that cured the case, but my general rule is to attend first to the most prominent acute symptoms that last appeared and then take up the older chronic trouble, or suppressed symptoms next. Since August entirely rid of the constipation; catamenia regular.

Case 5, November, 1897.—Charlotte, two years of age, had been delicate from birth. She had been taken ill at 7 in the morning. I saw her at 5.30 P. M., and was told by the mother that she commenced by crying and screaming as if with intense pain, but could not tell where. The symptoms were: Very restless, screaming, scolding, wanting everything she saw, and when getting it would refuse it or throw it at anybody near; would pull her hair and pinch her flesh anywhere. Would throw herself into all kinds of postures, as if in intense agony or in a rage of ill nature; must be carried all the time; had not eaten anything during the day. I watched her for ten minutes, and decided it to be a good case for Cham. I put a few pellets of the 50 M. in a dry teaspoon, and when she leaned over the mother's shoulder, with mouth wide open, screaming as if in intense agony, I threw the pellets into her mouth. The tension of her features relaxed, and she soon assumed a natural, quiet expression, and became still. The mother sat down, and in three minutes the child was on the floor playing and laughing with other children. She remained well, without any other remedy or another dose, until she had scarlet fever three months later with other children of the family.

Case 6.—Hemorrhage for eight years. This case I think worth attention on account of its history, as several of the most prominent gynecologists in the State had exhausted their surgical skill and science, and not only failed to relieve, but rather made matters worse.

Mrs. G., age 62, medium height, brown hair; naturally light complexion, but now bronzed; fleshy, weight about 160 pounds; mother of three grown children.

Saw her first July 17th, 1897.

Has had hemorrhage from the womb for eight years, never able to leave home, and often confined to bed many weeks at a time. Consulted the most eminent physicians and surgeons; was curetted twice with only temporary cessation of the hemorrhage, with other very distressing symptoms. When I first saw her, three months after the last curetting, she was suffering from vertigo—an unsteady feeling; could not trust the movement of her limbs, but must hold to the chair for fear of falling; had to hold to some one to walk or stand; could not walk in the dark; on turning head loses balance; sensation as if the bed was sinking under her; sensation as if hands were bent backwards; if hands hang down, arms pain like rheumatism.

I gave her Stram., 45 M., and S. L.

August 9th.—The sensorium much improved, but the hemorrhage increased. Sleeps better. Continued S. L.

August 20th.—But little improvement. Continued S. L.

September 4th.—Improvement stopped; hemorrhage worse. Pressing in vertex, all gone sensation in epigastrium at 11 A. M., and many other symptoms for Sulphur.

She got Sulph., M., and S. L.

September 18th.—Generally improved; continued S. L.

September 28th.—The Sulphur symptoms all gone, sensorium and hemorrhage no better. Gave Stram., CM., and S. L.

December 6th.—Sensorium much better, hemorrhage no better; blood, pale watery, with dark clots looking like burnt straw, and other symptoms which indicated Lachesis. Gave Lach., CM.

December 12th.—No better; very sick and vomiting. Gave Ipecac. 200. December 16th, Lach., MM.

December 23d.—No better, and I badly discouraged. No change in symptoms, the hemorrhage now the only trouble. She got Ovi-membrani Swan, DMM., and S. L.

December 26th.—Hemorrhage entirely stopped. It has not appeared since. No unfavorable symptoms following.

She now walks out and drives without inconvenience, which she has not done for eight years before.

I have had some wonderful effects from the Ovi-membrani in hemorrhages, but for want of provings have never used it in any other cases. In this case it must have been the true simillimum, for Lach., which appeared to be well indicated, did not do the work.

